# Vitamin D and chronic kidney disease: mechanisms, clinical implications, and future perspectives

**DOI:** 10.3389/fmed.2025.1643415

**Published:** 2025-10-14

**Authors:** Hong Wang, Tingting Yuan, Weihua Wu, Santao Ou

**Affiliations:** ^1^Department of Nephrology, The Affiliated Hospital of Southwest Medical University, Luzhou, Sichuan, China; ^2^Sichuan Clinical Research Center for Nephrology, Luzhou, Sichuan, China; ^3^Metabolic Vascular Disease Key Laboratory of Sichuan Province, Luzhou, Sichuan, China

**Keywords:** vitamin D, chronic kidney disease, vitamin D receptor protein, renal fibrosis, inflammation

## Abstract

**Background:**

Vitamin D deficiency is common in chronic kidney disease (CKD). Vitamin D/vitamin D receptor (VDR) signaling intersects inflammation, oxidative stress/mitochondrial injury, fibrogenic pathways, the renin–angiotensin–aldosterone system (RAAS), and the gut–kidney axis, providing a biologic rationale for renoprotection.

**Methods:**

Narrative review; literature identified from PubMed/MEDLINE, Embase, Web of Science, and Cochrane Library (January 2000–August 2025). Adult CKD populations (non-dialysis, dialysis, transplant) were included. Outcomes covered biologic/surrogate (e.g., proteinuria, estimated glomerular filtration rate [eGFR] slope) and hard endpoints (kidney failure, major cardiovascular events, fractures, mortality).

**Results:**

Nutritional vitamin D reliably corrects deficiency and improves laboratory profiles; VDR activators (VDRAs) suppress secondary hyperparathyroidism (SHPT). However, consistent benefits on hard outcomes have not been demonstrated across CKD settings, likely reflecting heterogeneity (baseline vitamin D status, stage, co-therapies, endpoints) and formulation/dosing differences (D₃ vs. D₂; cholecalciferol vs. calcifediol; steady vs. bolus). Safety considerations (hypercalcemia/mineral imbalance) apply to active agents and high-dose bolus regimens.

**Conclusion:**

A pragmatic approach is warranted: replete deficiency with nutritional vitamin D (prefer D₃; consider calcifediol when faster repletion or persistent SHPT is relevant), avoid mega-bolus dosing, and reserve active VDRAs for clear SHPT indications with careful calcium–phosphate–parathyroid hormone (PTH) monitoring—rather than positioning vitamin D as disease-modifying therapy for unselected CKD. Future trials should enrich truly deficient, higher-risk phenotypes, standardize regimens, and prioritize event-driven hard endpoints with embedded mechanistic markers to confirm on-target biology.

## 1 Introduction

Chronic kidney disease (CKD) represents a growing global health burden, with increasing mortality and disability-adjusted life years (DALYs) (1). Vitamin D deficiency is highly prevalent among patients with CKD and is closely associated with decreased renal function (2). The progressive loss of nephrons reduces renal 1α-hydroxylase activity, limiting the production of active 1,25-dihydroxyvitamin D₃ (1,25(OH)₂D₃), whereas urinary loss of vitamin D-binding protein (VDBP) due to proteinuria further exacerbates 25(OH)D deficiency (3). This dual impairment contributes not only to mineral metabolism disorders and secondary hyperparathyroidism but also to cardiovascular complications such as vascular calcification and left ventricular hypertropy (LVH) (4). Notably, vitamin D receptor (VDR) expression is widespread in renal tubular and vascular smooth muscle cells, and its signaling appears to exert both protective and potentially adverse effects (3, 5). Moreover, various forms of vitamin D compounds differ in their biological activities and clinical outcomes, complicating treatment strategies. Thus, a comprehensive review of the roles of vitamin D in CKD pathogenesis and its translational potential in disease progression and intervention is warranted.

## 2 Vitamin D metabolism and physiological functions

Vitamin D is a fat-soluble prohormone that is synthesized primarily in the skin from 7-dehydrocholesterol under ultraviolet B (UVB) radiation, forming previtamin D₃, which is then thermally isomerized into cholecalciferol (vitamin D₃). It can also be obtained from dietary sources in the form of either vitamin D₂ or D₃. Regardless of its origin, vitamin D undergoes two steps: first, in the liver, it is hydroxylated by 25-hydroxylase (CYP2R1) to form 25-hydroxyvitamin D₃ [25(OH)D₃], and subsequently, in the kidney, it is hydroxylated by 1α-hydroxylase (CYP27B1) to generate the biologically active form, 1,25-dihydroxyvitamin D₃ [1,25(OH)₂D₃] (6). In addition to renal activation, peripheral tissues such as immune cells also express CYP27B1, indicating that vitamin D can be locally activated to exert paracrine effects (7).

1,25(OH)₂D₃ exerts its effects by binding to the nuclear vitamin D receptor (VDR), forming a heterodimer with the retinoid X receptor (RXR), which then binds to vitamin D response elements (VDREs) on target genes. The ability of this complex depends on ligand-induced activation of both the VDR and RXR, reflecting a “dual-ligand requirement” for effective transcriptional regulation (8).

Physiologically, vitamin D plays a central role in calcium–phosphate homeostasis and bone metabolism. It enhances intestinal calcium absorption by upregulating calcium transport proteins (e.g., TRPV6, calbindin-D) and regulates calcium mobilization and reabsorption in bone and kidney tissues. Additionally, it indirectly modulates CYP27B1 expression through feedback regulation of parathyroid hormone (PTH) and fibroblast growth factor 23 (FGF23) (9).

In addition to mineral metabolism, vitamin D has immunomodulatory, anti-inflammatory, antioxidant, and potential antitumor effects. It suppresses Th17 cell responses, inhibits dendritic cell maturation, downregulates NADPH oxidase, and enhances glutathione antioxidant pathways to reduce reactive oxygen species and mitochondrial damage (10). Although preclinical studies suggest antitumor activity via modulation of the cell cycle, apoptosis, and angiogenesis, large-scale randomized controlled trials have not demonstrated a clear benefit in terms of cancer incidence or mortality (11).

**Figure 1:**
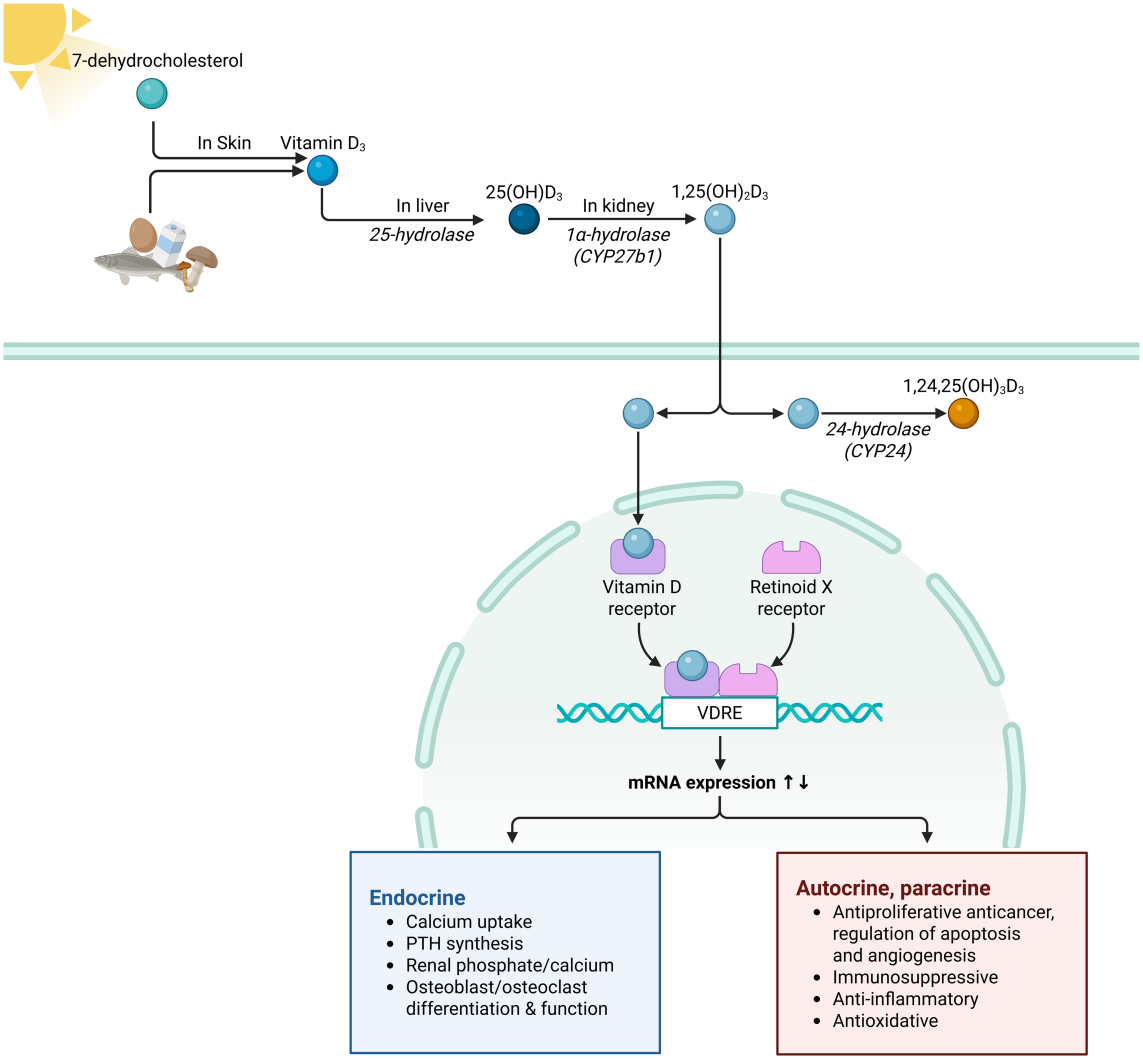
Vitamin D metabolism and physiological functions. This figure illustrates a brief overview of vitamin D’s physiological metabolism and functions. CYP24A1 = cytochrome P450 family 24 subfamily A member 1; CYP27B1 = cytochrome P450 ffamily 27 subfamily B member 1; VDRE = vitamin D response elements.

Overall, vitamin D metabolism involves coordinated actions across the skin, liver, kidney, and peripheral tissues. Its biological functions are mediated through VDR–RXR transcriptional control and are characterized by systemic relevance and context-dependent activity (Figure 1).

## 3 Renal regulation of vitamin D metabolism and its alterations in CKD

### 3.1 Physiological regulation of vitamin D metabolism in the kidney

The kidney plays a central role in the activation of vitamin D. In particular, proximal tubular epithelial cells express CYP27B1, which converts circulating 25-hydroxyvitamin D [25(OH)D] into its biologically active form, 1,25-dihydroxyvitamin D₃ [1,25(OH)₂D₃]. This hydroxylation step is tightly regulated by multiple endogenous factors. PTH enhances CYP27B1 expression via activation of the adenylyl cyclase–protein kinase A (AC–PKA) signaling pathway, thereby promoting the synthesis of active vitamin D (12). In contrast, FGF23 binds to the Klotho–FGFR complex and activates the Mitogen-Activated Protein Kinase/Extracellular signal-Regulated Kinase 1 and 2 pathway, leading to the suppression of CYP27B1 and upregulation of the catabolic enzyme 24-hydroxylase (CYP24A1), which accelerates the degradation of 1,25(OH)₂D₃ (13).

In addition to hormonal regulation, other metabolic signals also contribute to the control of vitamin D hydroxylase activity. Magnesium, as a cofactor for cytochrome P450 enzymes, enhances the catalytic function of CYP27B1. Inorganic phosphate can directly influence the expression of hydroxylases in tubular epithelial cells. Additionally, activation of the aryl hydrocarbon receptor (AHR) signaling pathway has been implicated in the transcriptional and possibly epigenetic regulation of both CYP27B1 and CYP24A1 (14, 15).

### 3.2 Dysregulation of vitamin D metabolism in chronic kidney disease

In the context of CKD, the regulatory network governing vitamin D metabolism becomes markedly disrupted. Inflammatory cytokines such as interleukin-6 (IL-6) and tumor necrosis factor-*α* (TNF-α), along with hypoxic stress, oxidative damage, and uremic toxins, have been shown to suppress the expression and activity of CYP27B1 through multiple signaling pathways, resulting in reduced synthesis of 1,25(OH)₂D₃ (16). Concurrently, persistent elevation of fibroblast growth FGF23 not only downregulates CYP27B1 but also induces the catabolic enzyme CYP24A1, accelerating the degradation of active vitamin D and further impairing its biological activity (13).

In addition to hydroxylase dysregulation, another critical barrier to effective vitamin D signaling in CKD lies in the impaired stability of the VDR. VDR expression is downregulated in CKD, and its protein turnover is tightly controlled by the ubiquitin-proteasome system. On one hand, 1,25(OH)₂D₃ can attenuate VDR ubiquitination and delay its degradation (17). On the other hand, certain stressors (e.g., uremic toxins) activate the AHR–Hsp90–MDM2 axis, promoting VDR ubiquitination and degradation (18), which aggravates local inflammation. In addition, activation of cyclin-dependent kinase CDK11p58 reduces VDR half-life via ubiquitin-mediated degradation, further weakening VDR’s transcriptional activity (19).

**Figure 2:**
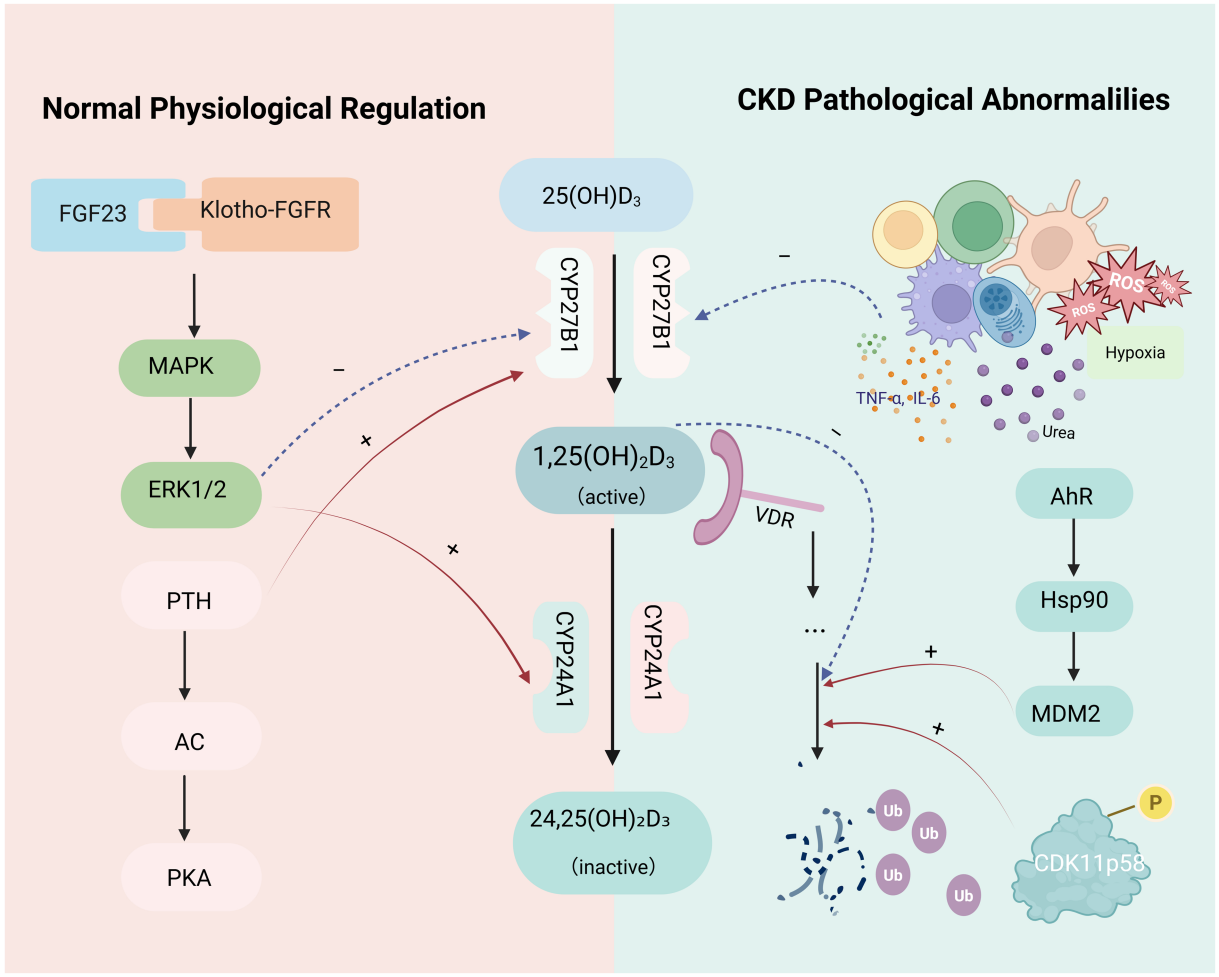
Renal regulation of vitamin D metabolism and its alterations in CKD. This figure illustrates the regulation of vitamin D metabolism by the kidneys. Solid arrows denote activation or promotion, while dashed arrows represent inhibition. AC = adenylate cyclase; AhR = aryl hydrocarbon receptor; CDK11p58 = cyclin-dependent kinase 11 p58; CYP24A1 = cytochrome P450 family 24 subfamily A member 1; CYP27B1 = cytochrome P450 family 27 subfamily B member 1; ERK1/2 = extracellular signal-regulated Kinase 1/2; FGF23 = fibroblast growth factor 23; Hsp90 = hheat shock protein 90; IL-6 = interleukin-6; MAPK = mitogen-activated protein kinase; MDM2 = mouse double minute 2; P = phosphorylation; PKA = protein kinase A; PTH = parathyroid hormone; ROS = reactive oxygen species; TNF-*α* = tumor necrosis factor-alpha; Ub = ubiquitination; VDR = vitamin D receptor.

Notably, renal transplantation provides direct evidence supporting the reversibility of vitamin D metabolic impairment. Studies have demonstrated that serum 1,25(OH)₂D₃ levels increase in CKD patients following kidney transplantation, indicating that the restoration of functional nephron mass can reestablish vitamin D activation capacity (20) (Figure 2).

## 4 The role of vitamin D in the pathogenesis of chronic kidney disease

The role of vitamin D in CKD extends beyond its classical function in calcium–phosphate homeostasis. Emerging evidence indicates that vitamin D, through receptor-mediated signaling, participates in multiple pathophysiological processes, including the regulation of immune and inflammatory responses, the mitigation of oxidative stress, the preservation of mitochondrial function, the inhibition of renal fibrosis, modulation of the renin–angiotensin–aldosterone system (RAAS), and the maintenance of gut–kidney axis homeostasis. This section provides a concise overview of these six aspects to elucidate the multifaceted roles of vitamin D in CKD pathogenesis and underscore its potential as a therapeutic target.

### 4.1 Immune and inflammatory response

Chronic inflammation and immune dysregulation are key drivers of CKD progression. Through activation of the VDR, vitamin D modulates both innate and adaptive immunity. It suppresses IL-6 and TNF-*α* production while enhancing IL-10 production in macrophages, and inhibits the mTOR/STAT3 pathway in T cells, thereby reducing proinflammatory Th17 cells and cytokines such as IL-17 and IL-22, while promoting regulatory T-cell (Treg) differentiation (21). Moreover, vitamin D upregulates TRAF3 expression to inhibit the noncanonical NF-κB2 pathway, alleviating the inflammatory state in end-stage renal disease (ESRD) patients and animal models (22). It also downregulates TLR4 and mitigates oxidative stress, reducing the release of monocyte-derived cytokines such as monocyte chemoattractant protein-1 (MCP-1) and TNF-*α* (23).

Clinically, paricalcitol supplementation has been shown to decrease serum IL-6 and TNF-α levels and reduce proteinuria in nondialysis CKD patients (24). More recent findings suggest that vitamin D may also improve hepcidin and serum iron profiles, indicating a potential link between inflammation and iron homeostasis (25).

Together, these findings support the role of vitamin D in modulating immune and inflammatory responses in CKD through multiple pathways and highlight its close connection to oxidative stress, which will be discussed in the next section.

### 4.2 Oxidative stress and mitochondrial dysfunction

Renal energy metabolism depends heavily on oxidative phosphorylation, and in CKD, mitochondrial dysfunction and oxidative stress form a vicious cycle that exacerbates tissue injury. Abnormalities in the mitochondrial electron transport chain (ETC), combined with impaired antioxidant defenses, lead to excessive accumulation of reactive oxygen species (ROS), which damage lipids, proteins, and mitochondrial DNA, ultimately promoting tubular apoptosis, interstitial fibrosis, and glomerulosclerosis (26, 27).

Vitamin D mitigates oxidative stress and mitochondrial damage through multiple mechanisms. On one hand, it activates the Nrf2 pathway via VDR, inducing the expression of antioxidant enzymes such as superoxide dismutase (SOD), GPX, and CAT to increase ROS scavenging, while simultaneously downregulating iNOS and NADPH oxidase to limit ROS production (23, 28). On the other hand, vitamin D helps stabilize the mitochondrial membrane potential, promotes ATP synthesis, and suppresses excessive mitochondrial fission, thereby maintaining mitochondrial integrity (29). It also enhances mitophagy and mitochondrial repair by modulating key autophagy regulators such as PTEN-induced kinase 1 (PINK1) and BCL2/adenovirus E1B 19 kDa protein-interacting protein 3 (BNIP3), and activating the Ca^2+^–CAMKK2–AMPK signaling pathway (30, 31).

Clinical and preclinical studies support these antioxidant effects. In ESRD patients, vitamin D supplementation significantly reduces serum malondialdehyde (MDA) and TNF-*α* levels, indicating improved oxidative status (32). In diabetic nephropathy models, VDR activation alleviates lipid peroxidation by modulating ATP citrate lyase (ACLY) and the Nrf2/Keap1 axis (29). Further studies suggest that vitamin D improves mitochondrial calcium homeostasis and protects against PM2.5-induced oxidative injury (33).

Overall, vitamin D exerts renoprotective effects in CKD by modulating ROS generation, enhancing antioxidant capacity, and preserving mitochondrial function, closely intersecting with its roles in immune and inflammatory regulation.

### 4.3 Renal fibrosis and extracellular matrix remodeling

Renal fibrosis is the common pathological endpoint of various chronic kidney diseases (CKD) and is characterized by fibroblast activation, epithelial-to-mesenchymal transition (EMT), and abnormal accumulation of the extracellular matrix (ECM), ultimately leading to nephron destruction and loss of function (34). The TGF-β1/Smad3 pathway is a key driver of this process, inducing the expression of fibrotic markers such as *α*-SMA and collagen I, promoting interstitial remodeling.

Vitamin D, which acts through VDR, modulates renal fibrogenesis at multiple levels. Studies have shown that vitamin D inhibits the activation of the TGF-*β*1/Smad3 signaling cascade by blocking Smad3 phosphorylation. Concurrently, it interferes with noncanonical pathways, including the Wnt/β-catenin and NF-κB pathways, thereby maintaining epithelial cell polarity and structural integrity and suppressing EMT and ECM deposition (35). In addition, vitamin D may exert indirect antifibrotic effects by upregulating bone morphogenetic protein-7 (BMP-7) or alleviating oxidative stress and inflammation (36).

The VDR agonist paricalcitol has demonstrated multitarget antifibrotic effects in experimental models. Deluque et al. reported that paricalcitol modulates the Angiopoietin-2/VEGF/VEGFR2 axis, attenuates TGF-*β*–related signaling, and preserves renal tissue architecture in an adriamycin nephropathy model (37). Similarly, Lim et al. showed that under combined hypoxia and TGF-β1 stimulation, paricalcitol inhibits Smad2 phosphorylation, regulates hypoxia-inducible factor-1 (HIF-1α) signaling, and reduces oxidative stress, thereby blocking the transition of pericytes into myofibroblasts (38).

In summary, vitamin D exerts renoprotective effects on renal fibrosis by suppressing profibrotic signaling, modulating multiple molecular pathways, and stabilizing the renal microenvironment. VDR agonists such as paricalcitol exhibit promising translational potential, although further studies are needed to clarify their mechanisms and clinical indications.

### 4.4 Activation of the renin–angiotensin-aldosterone system and disturbances in calcium–phosphate homeostasis

Aberrant activation of the RAAS is a critical driver of CKD progression, contributing to glomerular hypertension, inflammation, and fibrosis. Studies have shown that 1,25(OH)₂D₃ directly suppresses renin gene transcription in juxtaglomerular cells, thereby reducing angiotensin II levels and mitigating RAAS overactivation (39). Additionally, vitamin D inhibits the expression of RAAS-related receptors through VDR-mediated negative feedback mechanisms, indirectly exerting cardiorenal protective effects (40).

Disruption of calcium–phosphate metabolism is another hallmark of CKD, underlying the development of mineral and bone disorders chronic kidney disease–mineral and bone disorder (CKD-MBD), secondary hyperparathyroidism, and vascular calcification. Vitamin D regulates key transporters such as TRPV5 and NaPi-IIa in renal tubular epithelial cells, increasing calcium and phosphate reabsorption. It also modulates systemic mineral balance by suppressing parathyroid hormone (PTH) and fine-tuning the FGF23–Klotho signaling axis (41, 42), thus preserving calcium–phosphate homeostasis and bone–kidney axis integrity.

Taken together, these mechanisms illustrate vitamin D’s dual role: attenuating RAAS activation and correcting disordered mineral metabolism in CKD.

### 4.5 Gut–kidney axis regulation

The gut–kidney axis refers to the bidirectional interaction between the intestinal microbiota and the kidneys, which plays an increasingly recognized role in CKD progression (43, 44). As renal function declines, microbial diversity decreases, with a reduction in beneficial butyrate-producing bacteria and an expansion of uremic toxin–producing anaerobes such as indole- and p-cresol–generating strains (45). This dysbiosis, along with impaired intestinal barrier integrity, promotes endotoxin translocation and systemic inflammation (46).

Vitamin D, via the VDR regulates intestinal epithelial and mucosal immune cells, contributing to barrier maintenance and microbial homeostasis. It promotes the expression of tight junction proteins, enhances mucosal integrity, induces the antimicrobial peptide Reg3g, and modulates the activity of CD11b^+^ myeloid cells in Peyer’s patches. In humans, 1,25(OH)₂D₃ levels are positively associated with microbial *α*/*β*-diversity, particularly with the abundance of butyrate-producing Firmicutes (47).

Notably, microbial-derived metabolites themselves critically shape the gut–kidney interplay. Protein fermentation products such as indoxyl sulfate (IS), indole-3-acetic acid (IAA), and p-cresyl sulfate (PCS) accumulate in CKD and correlate with tubular interstitial fibrosis severity by activating the aryl hydrocarbon receptor (AHR) and NF-κB signaling (48). Trimethylamine-N-oxide (TMAO), another microbial metabolite, has been linked to oxidative stress, autophagy activation, and renal injury. By contrast, short-chain fatty acids (SCFAs), particularly butyrate, exert protective effects by engaging GPR41/43 and inhibiting histone deacetylases, thereby reinforcing epithelial barrier integrity and suppressing NF-κB/TGF-*β* pathways (49). Clinical metabolomic studies in peritoneal dialysis patients further demonstrate increased PCS and TMAO alongside reduced SCFAs, supporting the concept of a “toxin accumulation–SCFA depletion–barrier dysfunction–systemic inflammation” cascade (50).

In parallel, the microbiota may also influence vitamin D metabolism by modulating CYP27B1 expression, bile acid recycling, and SCFA production. Vitamin D, in turn, can affect renal inflammation and metabolic activity by sensing gut-derived metabolites such as SCFAs and TMAO through receptors including AHR and GPR41/43 (51). In summary, vitamin D acts not only as a regulator of mucosal immunity and microbial balance but also as an integrative node linking host, microbiota, and metabolite signaling within the gut–kidney axis, with promising implications for therapeutic intervention.

### 4.6 Other mechanisms

In addition to its classical roles, vitamin D influences several emerging pathways in CKD. Autophagy, a key cellular repair mechanism, is often impaired in early CKD, leading to cellular stress and injury. Calcitriol has been shown to enhance autophagic activity in podocytes and tubular cells by restoring LC3-II expression and autophagic flux, while concurrently reducing apoptosis through the modulation of Bcl-2 and Bax expression (21).

Klotho, an anti-aging factor predominantly expressed in the kidney, is downregulated in CKD and closely linked to vascular dysfunction. Vitamin D promotes Klotho expression and improves endothelial function, contributing to the regulation of the FGF23–Klotho axis and mineral balance (52).

In addition, vitamin D deficiency is associated with proteinuria and glomerular barrier damage. Supplementation has been linked to increased expression of slit diaphragm proteins such as nephrin and podocin, as well as reduced tubular injury, possibly through anti-inflammatory and anti-RAAS mechanisms (53).

**Figure 3:**
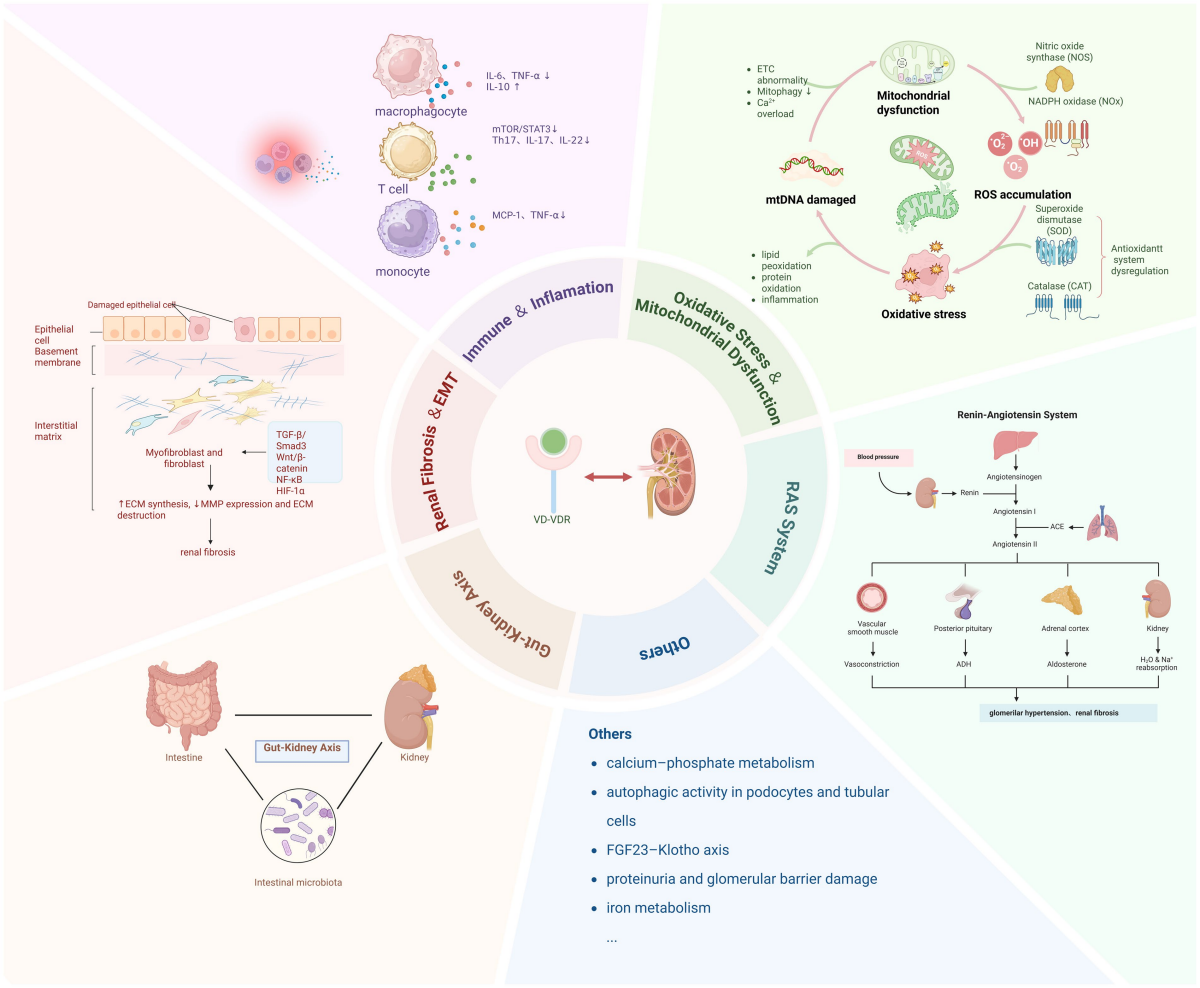
The role of vitamin D in pathogenesis of chronic kidney disease. This figure illustrates a brief overview of the role of vitamin D in the pathogenesis of chronic kidney disease. ACE = angiotensin-converting enzyme; CAT = catalase; ECM = extracellular matrix; ETC = electron transport chain; FGF23 = fibroblast growth factor 23; HIF-1α = hypoxia-inducible factor 1-alpha; IL-6 = interleukin-6; IL-10 = interleukin-10; MMP = matrix metalloproteinase; mTOR = mechanistic target of rapamycin; NF-κB = nuclear factor kappa-light-chain-enhancer of activated B cells; NOS = nitric oxide synthase; NOX = NADPH oxidase; RAAS = renin-angiotensin-aldosterone system; SOD = superoxide dismutase; STAT3 = signal transducer and activator of transcription 3; TGF-*β* = transforming growth factor-Beta; Th17 = T helper 17 cell; TNF-α = tumor necrosis factor-alpha.

Taken together, these findings suggest that vitamin D exerts renal protection in CKD not only through inflammation, oxidative stress, and fibrosis regulation but also via its roles in autophagy, Klotho signaling, and podocyte stability, highlighting its broader therapeutic potential (Figure 3).

## 5 Vitamin D in specific kidney diseases: mechanisms and clinical implications

### 5.1 Acute kidney injury

Acute kidney injury (AKI) is a rapidly developing clinical syndrome with poor outcomes, which affects approximately 20% of hospitalized patients (54). Recent evidence suggests that vitamin D is not only a potential biomarker for AKI risk but also may modulate AKI progression through multiple pathways.

Epidemiological studies have shown that low serum 25(OH)D levels significantly increase the risk of AKI and dialysis demand (55), whereas higher vitamin D status and sun exposure are independently associated with a lower incidence of AKI (56). Notably, 1,25(OH)₂D may be a more sensitive predictor of AKI and adverse outcomes in critically ill patients than 25(OH)D is (57).

Mechanistically, vitamin D exerts anti-inflammatory effects via VDR-mediated suppression of TLR signaling, induction of Treg expansion, and downregulation of proinflammatory cytokines such as TNF-*α* and IL-6. It also enhances antioxidant defenses by upregulating enzymes such as glutathione (GSH) and SOD and inhibits NADPH oxidase activity. In cisplatin and ischemia–reperfusion models, vitamin D protects tubular cells by downregulating ferroptosis-related molecules, including acyl-CoA synthetase long-chain family member 4 (ACSL4) and prostaglandin-endoperoxide synthase 2 (PTGS2) (55).

Clinically, multiple cohort studies support a causal link between vitamin D deficiency and AKI risk. Subgroup analysis of the VITDAL-ICU trial suggested that critically ill patients with a baseline 25(OH)D < 12 ng/mL may benefit from high-dose supplementation (58). However, some clinical studies highlight that excessive dosing may induce hypercalcemia and lead to prerenal or calcium deposition–related AKI, underscoring the need for precise dosing and indications (59, 60).

In summary, vitamin D may contribute to AKI prevention and recovery through immunomodulatory, antioxidant, and antiferroptotic mechanisms. Its therapeutic potential warrants further validation in large-scale randomized trials.

### 5.2 Diabetic kidney disease

Diabetic kidney disease (DKD), the most common microvascular complication of diabetes, affects approximately 40% of diabetic patients and remains a leading cause of end-stage renal disease worldwide (61). Despite advances in glycemic and RAAS-targeted therapies, the incidence and progression of DKD remain substantial, highlighting the need for additional therapeutic targets (62). Recent studies have focused on the pleiotropic effects of vitamin D on glucose metabolism, podocyte stability, inflammation, and fibrosis, suggesting its therapeutic relevance in DKD.

Mechanistically, vitamin D improves insulin sensitivity and *β*-cell function, contributing to glycemic control and mitigating microvascular damage at early stages (63). Podocytes, early targets of injury in DKD, exhibit downregulation of slit diaphragm proteins and increased apoptosis under hyperglycemic conditions. Active vitamin D has been shown to upregulate nephrin and podocin, restore autophagic flux, and reduce podocyte loss, thereby preserving glomerular barrier integrity (64).

Vitamin D also negatively regulates the advanced glycation end products (AGEs)–receptor for AGEs (RAGE) axis. In diabetic patients, supplementation reduces RAGE expression and the circulating levels of AGEs and TNF-*α*, potentially attenuating inflammation and fibrosis (65). Furthermore, it promotes renal lipid clearance through the activation of lipophagy, alleviating lipid-induced oxidative damage (66). These actions highlight the role of vitamin D in modulating DKD-specific metabolic and structural pathways in addition to its classical anti-inflammatory effects.

In clinical settings, multiple trials have demonstrated the benefits of vitamin D supplementation—whether in the form of active analogs or high-dose nutritional forms—in reducing proteinuria, improving inflammatory and metabolic profiles, and potentially stabilizing renal function in DKD patients (67–69). While the effects on eGFR remain variable, the overall trend supports a protective role in delaying structural and functional deterioration. In summary, vitamin D may exert multifaceted renoprotective effects in DKD through the modulation of glucose-lipid metabolism, podocyte biology, oxidative injury, and proinflammatory signaling, warranting further large-scale trials to refine its application in diabetic populations.

### 5.3 IgA nephropathy (IgAN)

IgA nephropathy (IgAN) is the most common primary glomerular disease, with treatment goals focused on reducing proteinuria and slowing the decline in renal function. Experimental studies suggest that vitamin D may attenuate renal injury by modulating immune responses and suppressing key inflammatory and fibrotic pathways, including the NF-κB/TLR4 and TGF-*β* signaling pathways (70). In clinical settings, several prospective and randomized studies have shown that supplementation with either active vitamin D or cholecalciferol can significantly reduce proteinuria, decrease the levels of inflammatory markers such as IL-6 and MCP-1, and increase VDR expression (70–75). Low serum vitamin D levels have also been associated with more severe histologic lesions and poorer prognosis in IgAN patients (76), and supplementation may improve cardiovascular autonomic function (77). Representative findings from clinical studies are summarized in Table 1.

**Table 1:** Clinical studies of vitamin D in IgA nephropathy.

Study (Year)	Study design	Intervention	Sample size	Main outcomes
Liu et al. (2012) (73)	Open-label RCT	Calcitriol 0.5 μg twice weekly × 48 wks	50	↓ proteinuria vs. control; ↑ VDR mRNA; safe profile
Xiaowei et al. (2020) (75)	RCT	Valsartan + Calcitriol 0.5 μg/day × 24 wks	303 (151 + 152)	98
Szeto et al. (2008) (71)	Uncontrolled prospective	Calcitriol 0.5 μg twice weekly × 12 wks	10	↓ proteinuria; ↓ TGF-β correlated with proteinuria
Deng et al. (2017) (74)	Meta-analysis	Calcitriol in non-nephrotic range proteinuria	6 RCTs, 384 patients	Calcitriol reduced proteinuria (WMD: −0.45 g/d, *p* < 0.00001)

RCT = randomized controlled trial; VDR = vitamin D;mRNA = messenger RNA; TGF-β = transforming growth factor beta; AEs = adverse events; WMD = weighted mean difference; wks = weeks.

Vitamin D may help reduce proteinuria and modulate immune responses in IgAN patients, but the current evidence is limited by the small sample size, short follow-up period, and various interventions. Most mechanisms are based on animal data, with little validation in humans. Future studies should focus on larger, longer trials and clarify dosing and target populations through mechanistic and subgroup analyses.

### 5.4 Lupus nephritis

Lupus nephritis (LN) is one of the most serious complications of systemic lupus erythematosus, and is driven by immune complex deposition, chronic inflammation, and progressive glomerular injury. While earlier animal studies reported inconsistent findings—such as potential proinflammatory effects of vitamin D in specific models (78)—more recent research has clarified its protective role in LN.

Experimental evidence suggests that vitamin D modulates key pathological pathways. It alleviates podocyte autophagy dysfunction by downregulating Beclin 1 and LC3B-II (79), reduces proteinuria and immune complex accumulation through inhibition of the NF-κB and MAPK signaling pathways (80), and blocks NLRP3 inflammasome activation via competitive binding to importin-4, thus preventing NF-κB nuclear translocation and improving renal structure and function (81). Clinical data further support these findings. In LN patients, low serum 25(OH)D levels correlate with increased soluble MHC Class I chain-related sequence A (sMICA) expression, reduced natural killer cel counts, and increased toll-like receptor 4 (TLR4) expression on T cells, indicating dysregulated innate immunity (82). In addition, vitamin D deficiency is associated with elevated urinary MCP-1, disrupted bone metabolism, and increased FGF23 levels, suggesting that vitamin D may influence LN progression through both immune and mineral-regulatory mechanisms.

Overall, vitamin D appears to exert anti-inflammatory, autophagy-preserving, and bone/mineral- regulating effects that may attenuate LN pathogenesis. However, key questions remain regarding the optimal dosing, timing of intervention, and patient-specific variability, warranting further high-quality prospective studies.

## 6 Vitamin D in CKD: timing and evidence

Over the past decade, large randomized trials in the general population have shown neutral effects of vitamin D on cancer, cardiovascular events, fractures, and all-cause mortality (83–88). Because CKD features prevalent vitamin D deficiency (VDD) and altered metabolism, such neutral findings should not be simply extrapolated. CKD-focused RCTs (Randomized Controlled Trials) indicate that in non-dialysis CKD, selective or non-selective VDRAs lower PTH but fail to improve LV mass/function or clinical hard outcomes and increase hypercalcemia risk (89, 90); in hemodialysis, oral alfacalcidol did not reduce cardiovascular composites or mortality (91), whereas nutritional vitamin D (cholecalciferol/calcifediol) raises 25(OH)D and improves biochemistry with neutral/insufficient evidence for hard outcomes so far (92–94). Accordingly, guidance endorses nutritional vitamin D as the base strategy to correct deficiency while avoiding mega-bolus dosing (≥100,000 IU) and excessive 25(OH)D; active VD/VDRAs are not for routine prevention in CKD G3–G5 (CKD stages 3–5) off dialysis but reserved for progressive/severe SHPT(secondary hyperparathyroidism) or PTH control on dialysis (95, 96). Kidney-transplant recipients are managed within the same “timing–formulation” pathway: prioritize safety and monitoring early post-transplant, replete if <30 ng/mL (75 nmol/L) thereafter—consider calcifediol when SHPT persists—and transition to CKD-style maintenance long term (96). Overall, RCTs underscore that biochemical gains do not necessarily translate into hard-outcome benefits, likely due to lack of true-deficiency enrichment, inadequate target attainment/duration, formulation/dosing differences (D3 > D2; faster attainment with calcifediol but requires monitoring), and pathway–endpoint mismatch; future trials should enroll deficient patients, use steady daily/weekly dosing, prioritize event-driven hard outcomes, and incorporate immune/epimeric measurements post-transplant to reduce exposure misclassification (97) (Table 2).

**Table 2:** Randomized controlled trials (RCTs) of Vitamin D—general population and CKD cohorts.

Population	Trial/Year	Sample & Setting	Intervention	Follow-up	Prespecified primary outcome	Main finding
General population	VITAL (Manson, 2019) (83)	US adults, ~25,000	Cholecalciferol 2000 IU/day	~5 years	Cancer, CVD	Neutral on hard outcomes
General population	D-Health (Neale, 2022; Thompson, 2023; Waterhouse, 2023) (84–86)	Australian adults, ~21,000	Cholecalciferol 60,000 IU/month	~5 years	All-cause mortality/MACE/Fracture	Neutral on primary outcomes; only exploratory signals
General population	WHI (Jackson, 2006; Hsia, 2007) (87, 88)	Postmenopausal women, ~36,000	Calcium + low-dose vitamin D	Long-term	Hip fracture / CVD	Neutral on primary outcomes
Non-dialysis CKD	PRIMO (Thadhani, 2012) (89)	CKD G3–G4 with LVH	Paricalcitol 2 μg/day	48 weeks	LV mass index	No difference; ↑ hypercalcemia
Non-dialysis CKD	OPERA (Wang, 2014) (90)	CKD G3–G5 with LVH	Paricalcitol 1 μg/day	52 weeks	LV structure/function	No difference; PTH ↓ (biochemical)
Hemodialysis	J-DAVID (2018) (91)	HD without marked SHPT	Alfacalcidol 0.5 μg/day	~4 years	CV composite/All-cause death	Neutral; ↑ biochemical Ca/P events
Hemodialysis	Morrone (2022) (92)	HD, multicenter	Oral calcifediol	24 months	All-cause/CV outcomes	Neutral; 25(OH)D ↑, PTH improved
Peritoneal dialysis	Brimble (2022) (93)	PD, factorial design	Cholecalciferol 50,000 IU/week ×8 → 10,000 IU/week ×44	52 weeks	CMR-measured LV mass	Neutral (underpowered); 25(OH)D ↑
Peritoneal dialysis (infection)	Zhang (2024, pilot RCT) (94)	PD, small randomized trial	Cholecalciferol 2000 IU/day	12 months	Time to recurrent peritonitis	Feasibility/attainment demonstrated; no efficacy signal

CKD = chronic kidney disease; HD = hemodialysis; PD = peritoneal dialysis; LVH = left ventricular hypertrophy; LV = left ventricle/left ventricular; CMR = cardiac magnetic resonance; CVD = cardiovascular disease; CV = cardiovascular; MACE = major adverse cardiovascular events; PTH = parathyroid hormone; SHPT = secondary hyperparathyroidism; Ca = calcium; P = phosphate; Ca/P events = calcium/phosphate-related laboratory events; 25(OH)D = 25-hydroxyvitamin D; VDRA = vitamin D receptor agonist; RCT = randomized controlled trial; CaD (WHI) = calcium plus vitamin D intervention; D3 = cholecalciferol; IU = international unit; y = year(s); mo = month(s); wk = week(s); G3–G5 = CKD stages 3–5; ↑ = increase; ↓ = decrease.

In sum, across populations and formulations, hard-outcome signals remain largely neutral: VITAL, D-Health, and WHI did not show clear benefits; in non-dialysis CKD, VDRAs lowered PTH without improving LV structure/function or clinical outcomes and posed hypercalcemia concerns; in dialysis, alfacalcidol was neutral for CV and mortality, while nutritional vitamin D corrected deficiency and improved labs but lacked definitive hard-outcome benefits. Consistent with Kidney Disease: Improving Global Outcomes (KDIGO) 2017—and reaffirmed by the 2024 KDIGO Controversies Conference—care should prioritize personalized management along bone (osteoporosis/fracture) and cardiovascular (calcification/left ventricular hypertrophy, LVH) axes: replete deficiency with nutritional vitamin D (D3 preferred; calcifediol when appropriate) using steady daily/weekly dosing with monitoring and avoiding mega-bolus; reserve active VD/VDRAs for clear SHPT indications (especially in dialysis). The Conference further underscores that vitamin D supplementation has no proven hard-outcome benefit in CKD, but this should not be misread as a reason to leave deficient patients untreated; in kidney-transplant recipients, RCT data suggest maintaining 25(OH)D ≥ 30 ng/mL may optimize bone endpoints (bone mineral density, BMD/fracture). PTH “targets” remain uncertain off dialysis; low-dose active VD can be an adjunct for PTH control, and extended-release calcifediol can suppress PTH at higher 25(OH)D (>125 nmol/L), though clinical-outcome data are still needed. (98)

Accordingly, future trials should enroll truly deficient, high-risk patients; directly compare D3 with modified-release calcifediol; prioritize event-driven hard outcomes—patient-important events such as all-cause death, MACE (major adverse cardiovascular events), kidney failure, or fracture, with follow-up continuing until a prespecified event count is reached—and incorporate immune and epimeric markers to link biologic responses with clinical endpoints.

## 7 Conclusion and future directions

As was previously stated, Vitamin D/VDR signaling touches many pathways in CKD. It tempers inflammation, oxidative stress, and fibrosis, and it modulates RAAS and the gut–kidney axis. These mechanisms make biological sense and support a renoprotective hypothesis. The clinical signal is less clear. Nutritional vitamin D corrects deficiency and improves laboratory profiles. Active analogues lower PTH. Yet consistent gains in hard outcomes—kidney failure, major cardiovascular events, or mortality—have not been shown. Evidence is heterogeneous across baseline vitamin D status, CKD stage, co-therapies, endpoints, and—crucially—formulation and dosing. For example, in non-dialysis CKD, paricalcitol did not reduce LV mass or improve cardiac function in PRIMO and OPERA despite biochemical effects, while in hemodialysis the J-DAVID trial found no reduction in cardiovascular events with oral alfacalcidol; by contrast, extended-release calcifediol reliably raised 25(OH)D and suppressed PTH in stage 3–4 CKD, but hard outcomes remain uncertain. Safety also requires attention, especially hypercalcemia and mineral imbalance with active agents or bolus regimens. A pragmatic approach is therefore warranted: replete deficiency with nutritional vitamin D (prefer D₃; consider calcifediol when faster repletion or persistent SHPT is relevant), avoid mega-bolus dosing, and reserve active VDRAs for clear SHPT indications with careful calcium–phosphate–PTH monitoring—not as disease-modifying therapy in unselected CKD.

Future work should be focused and lean: enroll truly deficient, higher-risk patients, standardize formulations and dosing, and test event-driven hard endpoints aligned to mechanism, while embedding translational markers to confirm on-target biology.
